# Mutational analysis of the *GNA11*, *MMP27*, *FGD1*, *TRRAP* and *GRM3* genes in thyroid cancer

**DOI:** 10.3892/ol.2013.1391

**Published:** 2013-06-11

**Authors:** AVANIYAPURAM KANNAN MURUGAN, CHONGFEI YANG, MINGZHAO XING

**Affiliations:** Laboratory for Cellular and Molecular Thyroid Research, Division of Endocrinology and Metabolism, The Johns Hopkins University School of Medicine, Baltimore, MD 21217, USA

**Keywords:** thyroid cancer, mutation, GNA11, MMP27, FGD1, TRRAP, GRM3

## Abstract

Frequent somatic mutations in the *GNA11*, matrix metalloproteinase (*MMP)27*, *FGD1, TRRAP* and *GRM3* genes have been reported in various types of human cancer, but whether these genes are mutated in thyroid cancer is not known. In the present study, a mutational analysis of these genes was performed in thyroid cancer cell lines and thyroid cancer samples. No *GNA11* mutations were identified in the papillary thyroid cancer (PTC), follicular thyroid cancer (FTC) and anaplastic thyroid cancer (ATC) samples. Additionally, no mutations were identified in the *MMP27* gene, although three synonymous [C351T (N117N), C1089T (S363S) and G1227A (G409G)] single nucleotide polymorphisms (SNPs) were observed infrequently in ATC. No mutations were detected in the *FGD1* gene, but two infrequent synonymous [T2091C (T697T) and A2136G (P712P)] SNPs were observed in PTC. Furthermore, no mutations were identified in *TRRAP* and *GRM3*, although a frequent synonymous SNP [G1323A (T441T)] and infrequent non-synonymous SNP [G1424A (G475D)] of *GRM3* were observed in PTC. No mutation of these genes was observed in 12 cell lines derived from various types of thyroid cancer. The present study reports for the first time the mutational status of the *GNA11, MMP27, FGD1, TRRAP* and *GRM3* genes in thyroid cancer. No mutations were identified in these genes in the various types and cell lines of thyroid cancer. Therefore, unlike in other types of cancer, mutations in these genes are absent or uncommon in thyroid cancer.

## Introduction

Thyroid cancer is the most common endocrine malignancy with a high incidence in numerous regions of the world (1,2). Thyroid cancer is histologically classified into papillary thyroid cancer (PTC), follicular thyroid cancer (FTC) and anaplastic thyroid cancer (ATC). Thyroid cancers frequently harbor activating mutations in the mitogen-activated protein kinase (MAPK) and phosphatidylinositol 3-kinase (PI3K)/Akt signaling pathways, as represented by *RET/PTC, RAS* and *BRAF* mutations in the former and by *PIK3CA* and *PTEN* mutations in the latter (3,4). As a significant mechanism for the tumorigenesis of thyroid cancer, aberrant activation of these two important signaling pathways by such mutations causes uncontrolled cell division, proliferation and survival, leading to malignancy.

High-frequency somatic mutations of the *GNA11,* matrix metalloproteinase (*MMP)27, TRRAP* and *GRM3* genes have been reported in uveal melanomas and melanomas with various incidences (5–8). We previously demonstrated that *FGD1* was normally maintained, hypomethylated and overexpressed by *BRAF* (V600E) in thyroid cancer cells (9). GNA11 activates the MAPK signaling pathway. Particularly frequent somatic mutations of the *GNA11* gene at codon 209 in exon 5 and codon 183 in exon 4, resulting in mutant GNA11^Q209L^ and GNA11R183C, respectively, have been reported in uveal melanoma and blue nevi (5). The *GNA11* gene encodes a G-protein α-subunit (Gα11) that mediates signals from G-protein-coupled receptors (GPCRs) to the MAPK pathway. The normal amino acid, glutamine, encoded by codon 209 of the *GNA11* gene, lies within the RAS-like domain of GNA11 (corresponding to residue 61 of Ras) and is essential for GTP hydrolysis. In members of the RAS family, mutations at this site and at codon 12 cause the loss of GTPase activity with constitutive activation of Ras. The GNA11^Q209L^ and GNA11R183C mutants have been demonstrated to be able to transform 3T3 cells and form tumors in immunocom-promised mice (5). MMPs are proteolytic enzymes that degrade components of the extracellular matrix and basement membranes. MMP abnormalities have been associated with the metastasis of various types of cancer (6,10–12). In particular, mutations of the *MMP27* gene have been observed in melanoma. The majority of the mutations in this gene have been identified in exons 1, 2, 3, 8 and 9 (6). *FGD1* gene mutations have been reported in Aarskog-Scott syndrome (AAS), or facio-digito-genital dysplasia (13). At present, 20 different FGD1 gene mutations have been reported in this syndrome (13). *FGD1* is a Dbl family member that has been shown to function as a CDC42-specific guanine nucleotide exchange factor (GEF). It has also been demonstrated that FGD1 expression is sufficient to cause tumorigenic transformation of NIH3T3 fibroblasts (14). Two studies from the same group reported that the *TRRAP* gene was recurrently mutated and clustered in one amino acid position S722F (7). Furthermore, a frequent mutation of the *GRM3* gene has been reported, and the authors also noted that the mutant selectively regulated the phosphorylation of MEK in the activation of the MAPK signaling pathway, leading to the anchorage-independent growth and migration of cells (8). The mutation status in the *GNA11*, *MMP27*, *FGD1*, *TRRAP* and *GRM3* genes has not been studied in thyroid cancer. The present study was conducted to investigate the mutational status of these genes in thyroid cancer.

## Materials and methods

### Cell lines, tumor samples and DNA extraction

A total of 89 samples, consisting of 12 thyroid cancer cell lines and 77 thyroid tumor samples were used for the mutational analysis of the *GNA11* gene. For the *MMP27* mutational analysis, 29 samples consisting of 12 thyroid cancer cell lines and 17 ATC samples were used. The *FGD1*, *TRRAP* and *GRM3* genes were analyzed in 28 samples, including 12 thyroid cancer cell lines and 16 PTC samples. The thyroid cancer cell lines and tumor samples were used as described previously and with institutional review board (IRB; The Johns Hopkins University School of Medicine, Baltimore, MD, USA) approval (15). The cell lines were authenticated as described previously (16). With the exception of the FTC133 cells cultured in Dulbecco’s modified Eagle’s medium (DMEM)/Ham’s F-12 medium, all tumor cell lines were cultured in RPMI-1640 medium supplemented with 10% fetal bovine serum (FBS), streptomycin (100 μg/ml), penicillin (100 U/ml) and 2 mM glutamine. Genomic DNA from the cell lines and tumors was isolated by standard phenol-chloroform extraction using MaXtract high-density gel tubes followed by ethanol precipitation procedures (Qiagen, Valencia, CA, USA) (15).

### PCR amplification and sequencing of the GNA11, MMP27, FGD1, TRRAP and GRM3 genes

The primer sequences and PCR conditions for the amplification of exons 4 and 5 of the *GNA11* gene and exons 1, 2, 3, 8 and 9 of the *MMP27* gene were as described previously (5,6). For the mutational analysis of the *MMP27* gene, in addition to the above primers, additional primers were used to shorten the amplicon size. These primers are shown in Table I. The PCR conditions for the amplification of the *MMP27* with the additional primers were as follows: One cycle of 94°C for 3 min; ten cycles of 94°C for 30 sec, 67°C for 30 sec with a 1°C reduction for each cycle and 72°C for 30 sec; thirty two cycles of 94°C for 30 sec, 57°C for 30 sec and 72°C for 30 sec; followed by 72°C for 7 min as a final extension; and 4°C as the storage temperature. The primer sequences for the amplification of the *FGD1* gene are shown in Table I. The PCR reaction conditions for the *FGD1* amplification were as follows: After an initial denaturation at 94°C for 2 min, the amplification was performed at 94°C for 1 min, followed by annealing temperatures (exons 11, 14 and 18, 55°C; exons 1, 2 and 16, 57°C; exons 5, 6, 7, 8, 13 and 17, 58°C; exons 3, 12 and 15, 60°C; and exons 4, 9 and 10, 62°C) for 1 min for 35 cycles, with a final extension at 72°C for 7 min. The primer sequences and PCR conditions for the amplification of exon 1 of the *TRRAP* gene and exons 1, 2, 3, 4 and 5 of the *GRM3* gene were as described previously (7,8). The PCR products were directly sequenced using a BigDye Terminator v3.1 Cycle Sequencing ready reaction kit (Applied Biosystems, Foster City, CA, USA). These exons were investigated as they harbored the majority of the reported mutations in these genes in human cancers. The GenBank accession numbers were NM_002067.2 (*GNA11*), NM_022122 (*MMP27*), NM_004463 (*FGD1*), NM_003496 (*TRRAP*) and NM_000840 (*GRM3*).

## Results

The strategy of the present study was to investigate the gene exons that were the most likely to carry mutations. In particular, exons 4 and 5 of the *GNA11* gene were examined for mutations since all of the known *GNA11* mutations have been reported in codons 209 and 183 of these exons. Exons 1, 2, 3, 8 and 9 of the *MMP27* gene were selected for sequencing as they have been shown to carry somatic mutations in melanomas. All the exons of the *FGD1* gene were analyzed for mutation, as mutations in this gene have never been reported in human cancers. Exon 1 of the *TRRAP* gene and exons 1, 2, 3, 4 and 5 of the *GRM3* gene were analyzed as these exons have also been reported to harbor somatic mutations in melanoma.

**Figure 1.**
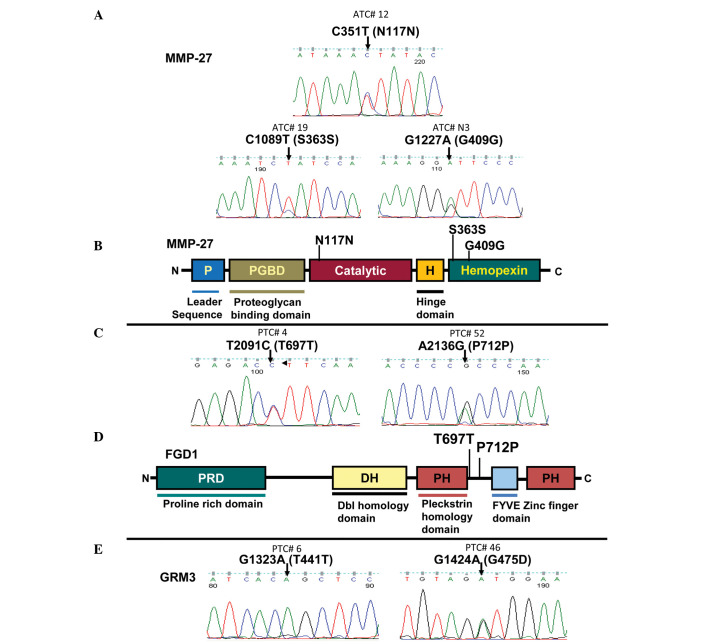
Detection of MMP27, FGD1 and GRM3 mutations. (A) Sequencing results are shown with a representative sense sequence profile of SNPs (N117N, S363S and G409G) detected in exons 3, 8 and 9 of the *MMP27* gene. Arrows indicate mutated nucleotides. (B) Schematic diagram of domains of the MMP27 protein showing three SNPs (N117N, S363S and G409G) identified in thyroid cancer. (C) Sequencing results are shown with a representative sense sequence profile of SNPs (T697T and P712P) detected in exon 14 of the *FGD1* gene. Arrows indicate mutated nucleotides. (D) Schematic diagram of domains of the FGD1 protein showing two SNPs (T697T and P712P) identified in thyroid cancer. (E) Sequencing results are shown with a representative sense sequence profile of two SNPs (T441T and G475D) detected in exon 3 of the *GRM3* gene. Arrows indicate mutated nucleotides. The nucleotide, amino acid alteration and tumor number are indicated above the arrows. Nucleotide numbers refer to the position within the coding sequences, where position 1 corresponds to the first position of the initiation codon. All the samples were sequenced in two repeated examinations with independent PCR by forward and reverse primers. MMP, matrix metalloproteinase; SNPs, single nucleotide polymorphisms.

The sequencing results showed no mutations in and around the hot spot of codons 209 and 183 in the *GNA11* gene in 12 thyroid cancer cell lines and 46 thyroid cancer samples (including 26 FTC and 20 ATC samples). No novel *MMP27* somatic mutations were identified in 12 thyroid cancer cell lines and 15 ATC tumor samples. As shown in Fig. 1A and B, an infrequent [1/17 (5.8%)] C>T transition was observed at nucleotide position 351, resulting in a codon change of AAC>AAT and amino acid N117N in exon 3. In exon 8, an infrequent [1/17 (5.8%)] C>T transition was observed at nucleotide position 1089, resulting in a codon change of TCC>TCT and amino acid S363S. In exon 9, a frequent [7/17 (41.2%)] G>A transition at nucleotide position 1227 was also observed, resulting in a codon change of GGG>GGA and amino acid G409G. The two N117N and S363S mutations were rare and novel silent mutations that have not been previously reported in the SNP database.

No *FGD1* mutations were identified in 12 thyroid cancer cell lines. However, as illustrated in Fig. 1C and D, an infrequent [1/16 (6.3%)] T>C mutation was observed at nucleotide position 2091, resulting in a codon change of ACT>ACC and amino acid T697T. An infrequent [1/16 (6.3%)] A>G was also observed at nucleotide position 2136, resulting in a codon change of CCA>CCG and amino acid P712P. These silent T697T (rs12011120) and P712P (rs1126744) mutations were rare SNPs that have been reported in the SNP database (http://www.ncbi.nlm.nih.gov/projects/SNP/).

Mutations were not identified in the *TRRAP* gene in 12 thyroid cancer cell lines and 16 PTC tumor samples. No *GRM3* mutations were detected in 12 thyroid cancer cell lines. A G>A mutation was observed at nucleotide position 1323, resulting in a codon change of ACG>ACA and amino acid T441T in all 12 thyroid cancer cell lines and 16 PTC samples. An infrequent [1/16 (6.3%)] G>A mutation was also observed at nucleotide position 1424 resulting in a codon change of GGT>GAT and amino acid G475D (Fig. 1E). The T441T mutation was a novel synonymous SNP that has not previously been reported in the SNP database. G475D (rs17161026) was a non-synonymous SNP that has previously been reported in the SNP database (http://www.ncbi.nlm.nih.gov/projects/SNP/). Fig. 1 shows the mutations identified in the present study, and a summary of the results is presented in Table II.

## Discussion

In the present study, several genes were analyzed for the first time for possible mutations in thyroid cancer. *GNA11* mutations were analyzed in all types of thyroid cancer (PTC, FTC and ATC) as they have been frequently identified in uveal melanoma and are known to activate the MAPK signaling pathway (5), which is one of the most deregulated signaling pathways in thyroid cancer (3). However, no mutations were detected in and around codons 209 and 183. These two hot spot codons were selectively analyzed as GNA11 mutations have consistently been identified only in these two residues (5). The MMPs are proteolytic enzymes that degrade the components of the extracellular matrix and basement membranes, which are associated with cancer metastasis (6,10–12). ATC is the most aggressive type of thyroid cancer that is often associated with deadly metastasis (17). Therefore ATC was particularly analyzed for the mutation of the *MMP27* gene. Two *MMPs* have been reported to occasionally be mutated in melanomas. The *MMP27* gene was analyzed for mutation in ATC in the present study as we had already previously analyzed the second gene, *MMP8,* in thyroid cancer (18). Three uncommon mutations were identified; C351T resulting in N117N, C1089T resulting in S363S and G1227A resulting in G409G silent mutations. The silent mutations are unlikely to be involved in thyroid carcinogenesis as these mutations do not change the basic amino acids.

We previously revealed that *FGD1* was normally maintained, hypomethylated and overexpressed by *BRAF* (V600E) in thyroid cancer cells and in turn observed that it was hyper-methylated after ShRNA-mediated knockdown of *BRAF* (V600E) in thyroid cancer cell lines (9). Based on these findings and the high transforming and invasive potential of the *FGD1* gene (4), we considered there to be a high possibility of identifying oncogenic mutations in *FGD1*. All 18 exons of the gene were sequenced to be analyzed for mutations, but only two silent mutations (T697T and P712P) were detected. These mutations are unlikely to have a significant role in PTC. No somatic missense mutations were identified in the *FGD1* gene.

The *TRRAP* gene has been reported to be mutated in a particular codon, S722F (7). As GRM3 activates the MAPK signaling pathway (8), the present study investigated whether *GRM3* is mutated in PTC samples, since the majority of PTCs harbor genetic deregulation in the MAPK signaling pathway. No mutations were detected in TRRAP, while two SNPs (T441T and G475D) were identified in the *GRM3* gene. This suggests that *TRRAP* and *GRM3* may not have important roles in the pathogenesis of this type of thyroid cancer.

In conclusion, the present findings suggested that genetic alterations in the *GNA11, MMP27, FGD1, TRRAP* and *GRM3* genes may not be significant in the tumorigenesis of thyroid cancer. It is not surprising that mutations in these genes are not common in thyroid cancer since a number of the upstream effectors involved in cellular transformation, growth and metastasis, including EGFR, RET/PTC, ALK, RAS, BRAF, PTEN, PIK3CA, PIK3CB and PDK1, are commonly genetically altered via mutations or genetic amplifications that are able to independently activate the MAPK or PI3K/Akt pathways in thyroid cancer (3,4,16,18,19).

## Figures and Tables

**Table I.** Primer sets used for PCR amplification of the *MMP27* and *FGD1* genes.

Exon	Forward	Reverse
*MMP27*		
1-1	GCCAATTCATGCCACGTCTCAC	GAAAATATGCAACTGGCTCAGG
1-2	GAACCGGCTTCAGCTGAAGAAAG	CACATTCCTGCAAAAGAGTCCTG
2-1	CCTGAGATGGAGATTTGCTCTC	GGATTGACAGTGACTGGAAAAC
2-2	TCTTTTTGGTCAGGCATATCTC	ATCATG AAGACACCCAGGTGTG
2-3	CTCAACCAGT TCTACTCTCTTG	GTATGGCTACACCCTCCCT
2-4	TGAGATCATG AAGACACCCAGG	CATTCAATTGACTGAGCACTTC
*FGD1*		
1	GGCTTGAGTCTCTGCAGTG	AACAAGAACCCGCTCCCAGTAC
2	AGTCCTAACTTTAACCCCAGTC	CTGGCTAACTTCTCCCCTCCTC
3	TTCACCATGTTAGCCAGGCTC	GTATGAGCTTGACTGAGAGGC
4	AGCCTGGGACAGGAAGGGATA	AAAGGCGCTTCCAGGTTCTCC
5	TATTAGGCTTAGAGTGGCATG	ACTGCCTCCTTGAAACGCACC
6	TCAGTCTCAAGACCAATGCTG	GAAGTCTTGTGTACACCTCTG
7	ACTGAGATGAAAGGTATCTGC	TCAGATCTGGCTGCAGATGCC
8	AAGCTGGAAGGAGCAGACTTG	AGAGCTATTAGTGTGGAGAAG
9	AGGCAGAGGTTGTGGTGAGCC	GTGCCAGCCTCCTGTCAGATG
10	ATCTGACAGGAGGCTGGCACC	AGTACCAGGTCACTATGTGTG
11	CAGTAAAGCTTCAGGGCAAG	AGCCAGCATCTTTGTTCCTC
12	TGTGTGTGTATGTGTGCAGAG	TCTCTGGGCCTGGAATGCCTC
13	AGGTGAAGAAGAGGGTCAAGC	ACCATTCTGGTTAGCTGTGAG
14	CTAGGGTATACGAAGGTGAGGC	TAAAGGTCAGGTGGGCATTTGG
15	ATGATAATCCAAGCGTTGGAG	AGGGTACCCACTCTGCAGTGG
16	TGCTGTGGGAGTTGGTATGCTG	TGCTGTGGGAGTTGGTATGCTG
17	TTCATCTGAGATAGGAATAGC	TCTTCCAAGGCTCACCTTATC
18	GCAAAACCAGTTAGAAGCTGGG	TTCAAGTATTGACTGAGCTGGG

Primers are represented as 5′>3′. MMP, matrix metalloproteinase.

**Table II.** Mutations of the *MMP27*, *FGD1* and *GRM3* genes in thyroid cancers.

S. No.	Gene	Exon	Nucleotide	Codon	Amino acid	Type of mutation
ATC# 12	*MMP27*	3	C351T	AAC-AAT	N117N	Silent
ATC# 19	*MMP27*	8	C1089T	TCC-TCT	S363S	Silent
ATC# N3	*MMP27*	9	G1227A	GGG-GGA	G409G	Silent
PTC# 4	*FGD1*	14	T2091C	ACT-ACC	T697T	Silent
PTC# 52	*FGD1*	14	A2136G	CCA-CCG	P712P	Silent
PTC# 6	*GRM3*	3	G1323A	ACG-ACA	T441T	Silent
PTC# 46	*GRM3*	3	G1424A	GGT-GAT	G475D	Missense

MMP, matrix metalloproteinase.
